# Autofluorescence Imaging of Treatment Response in Neuroendocrine Tumor Organoids

**DOI:** 10.3390/cancers13081873

**Published:** 2021-04-14

**Authors:** Amani A. Gillette, Christopher P. Babiarz, Ava R. VanDommelen, Cheri A. Pasch, Linda Clipson, Kristina A. Matkowskyj, Dustin A. Deming, Melissa C. Skala

**Affiliations:** 1 Department of Biomedical Engineering, University of Wisconsin, Madison, WI 53706, USA; agillette@wisc.edu; 2 Department of Medicine, Division of Hematology, Oncology and Palliative Care, School of Medicine and Public Health, University of Wisconsin, Madison, WI 53705, USA; babiarz2@wisc.edu; 3 Morgridge Institute for Research, Madison, WI 53715, USA; avandommelen@wisc.edu; 4 University of Wisconsin Carbone Cancer Center, Madison, WI 53705, USA; capasch@uwcarbone.wisc.edu (C.A.P.); matkowskyj@wisc.edu (K.A.M.); 5 McArdle Laboratory for Cancer Research, Department of Oncology, University of Wisconsin, Madison, WI 53705, USA; lclipson@wisc.edu; 6 Department of Pathology and Laboratory Medicine, University of Wisconsin, Madison, WI 53705, USA

**Keywords:** fluorescence lifetime imaging, NAD(P)H, autofluorescence, neuroendocrine tumor, organoid

## Abstract

**Simple Summary:**

Gastroenteropancreatic neuroendocrine tumors (GEP-NET) account for roughly 60% of all neuroendocrine tumors, and low/intermediate grade human GEP-NETs have relatively slow growth rates that many laboratory culture methods fail to capture. Patient-derived cancer organoids (PDCOs) are an attractive model to address this need for relevant 3D cultures of GEP-NETs for laboratory drug testing. However, traditional measurements of drug response are not effective in GEP-NET PDCOs due to the small volume of tissue and slow growth rates that are characteristic of the disease. Here, we test a label-free, non-destructive optical metabolic imaging (OMI) method to measure drug response in live GEP-NET PDCOs. OMI measured a response to the novel treatment combination of ABT-263 and everolimus in five out of seven PDCO lines, at 72 h post-treatment. Overall, this work shows that OMI provides single-cell metabolic measurements of drug response in PDCOs to guide drug development for GEP-NET patients.

**Abstract:**

Gastroenteropancreatic neuroendocrine tumors (GEP-NET) account for roughly 60% of all neuroendocrine tumors. Low/intermediate grade human GEP-NETs have relatively low proliferation rates that animal models and cell lines fail to recapitulate. Short-term patient-derived cancer organoids (PDCOs) are a 3D model system that holds great promise for recapitulating well-differentiated human GEP-NETs. However, traditional measurements of drug response (i.e., growth, proliferation) are not effective in GEP-NET PDCOs due to the small volume of tissue and low proliferation rates that are characteristic of the disease. Here, we test a label-free, non-destructive optical metabolic imaging (OMI) method to measure drug response in live GEP-NET PDCOs. OMI captures the fluorescence lifetime and intensity of endogenous metabolic cofactors NAD(P)H and FAD. OMI has previously provided accurate predictions of drug response on a single cell level in other cancer types, but this is the first study to apply OMI to GEP-NETs. OMI tested the response to novel drug combination on GEP-NET PDCOs, specifically ABT263 (navitoclax), a Bcl-2 family inhibitor, and everolimus, a standard GEP-NET treatment that inhibits mTOR. Treatment response to ABT263, everolimus, and the combination were tested in GEP-NET PDCO lines derived from seven patients, using two-photon OMI. OMI measured a response to the combination treatment in 5 PDCO lines, at 72 h post-treatment. In one of the non-responsive PDCO lines, heterogeneous response was identified with two distinct subpopulations of cell metabolism. Overall, this work shows that OMI provides single-cell metabolic measurements of drug response in PDCOs to guide drug development for GEP-NET patients.

## 1. Introduction

Gastroenteropancreatic neuroendocrine tumor (GEP-NET) incidence and prevalence have increased 6-fold over the last 30 years [1]. This elevated incidence is likely due to increased use of CT scan screening for other reasons, which enables incidental detection of GEP-NETs. Unresectable well-differentiated GEP-NETs are generally considered incurable, and options for systemic treatment are limited, particularly after progression on somatostatin analogues and peptide receptor radionuclide therapy (PRRT). Cytotoxic chemotherapy remains an option to extend lifespan for some patients, although with a high burden of toxicity [2]. Therefore, improved treatment options for GEP-NET patients are urgently needed. The mammalian target of rapamycin (mTOR) pathway is upregulated in some GEP-NETs and can be targeted with the mTOR inhibitor everolimus. Although large phase 3 trials have proven the efficacy of everolimus monotherapy in treating pancreatic and non-pancreatic GEP-NETs, most patients will have progressive disease after less than 1 year of treatment [3,4]. Further, the best response for the majority of GEP-NET patients treated with everolimus was stable disease [3,4]. Given the moderate effect of everolimus in these phase 3 trials, one strategy is to combine everolimus with other therapies in a rational manner.

Proposed mechanisms of resistance to mTOR inhibitors include compensatory activation of other oncogenic pathways and upstream activation of the PIK3CA-AKT-mTOR pathway [5]. Importantly, the PIK3CA-AKT-mTOR pathway is a regulator of the anti-apoptotic Bcl-2 family of proteins, which in turn regulate mitochondrial apoptosis [6]. Additionally, AKT activation has been shown to increase Bcl-2 expression [7]. Thus, cells exposed to mTOR inhibitors may be more dependent on Bcl-2 for survival, and more sensitive to therapeutics that inhibit the function of the Bcl-2 family, supporting a synergistic effect for the combination of mTOR and Bcl-2 inhibition [8]. This proposed synergism between mTOR inhibitors and Bcl-2 inhibitors has been confirmed in pre-clinical studies of small cell lung cancer and leukemia [9,10].

Pre-clinical drug screens are difficult to perform for GEP-NETs because there are currently no readily available cell lines or animal models that reliably recapitulate well-differentiated human GEP-NETs. These challenges in pre-clinical modeling are due to the relatively low proliferation of human GEP-NETs [11,12], the expression of neuroendocrine markers, and the unique genetic drivers that are not always maintained in cell culture models [13]. Short-term patient-derived cancer organoids (PDCOs) are a model system that holds great promise for recapitulating well-differentiated human GEP-NETs [14,15,16]. PDCOs are three-dimensional cultures of a patient-matched tumor grown in a 3D matrix. Previous studies have established that patient-derived cell culture of GEP-NET samples recapitulate key features of the original patient tumor, including driver mutations, surface markers, proliferation, and drug response [17,18].

The small volume of tissue and a slow proliferation rate of GEP-NET PDCOs limits the relevance of standard measurements of PDCO drug response (i.e., growth, proliferation), which rely on accelerated growth and often require sample destruction. Therefore, metabolic imaging methods are attractive to monitor treatment response in GEP-NET PDCOs. Previous imaging-based studies have investigated cell metabolism in 3D PDCO cultures with optical probes that measure intracellular oxygen [19], glucose concentrations [20], and imaging mass spectrometry for metabolite profiling [21]. Here, two-photon optical metabolic imaging (OMI) of GEP-NET PDCOs is used in this study to monitor drug response on a single-cell level within intact samples. OMI is a non-destructive, high-resolution 3D microscopy technique that measures cell metabolism based on the autofluorescence of co-enzymes NAD(P)H and FAD. The fluorescence properties of NADH and NADPH overlap and are jointly referred to as NAD(P)H. However, it is important to note that they do play different roles in metabolic pathways, and while the NADH/NAD+ ratio is generally less oxidized in cells compared to the NADHPH/NADP+ ratio, the ratios are coupled. This suggests that changes in the combined NAD(P)H autofluorescence can be used to monitor for changes in the reduced state of the individual cofactors [22,23,24]. The optical redox ratio, defined as the ratio of the fluorescence intensity of NAD(P)H to that of FAD, reflects the redox state of the cell, as NAD(P)H is an electron donor while FAD is an electron acceptor [25,26]. The fluorescence lifetimes of NAD(P)H and FAD are both bi-exponential with distinct lifetimes for the free and protein-bound conformations, and thus reflect the protein-binding activities of NAD(P)H and FAD [27,28]. The mean lifetime (τ_m_) is the weighted average of the short and long lifetimes (τ_1_, τ_2_, respectively) from the bi-exponential fit. The optical redox ratio, along with NAD(P)H and FAD τ_m_, provide complementary information that can be used to interpret the metabolic state of cells [26]. The OMI variables (normalized redox ratio, NAD(P)H τ_m_, FAD τ_m_) can be linearly combined (coefficients 1, 1, −1, respectively) to form a composite OMI index. The OMI index is a metabolic measure of drug response that precedes changes in cell viability or tumor size [29]. Generally, cellular rates of glycolysis, and NAD(P)H and FAD protein-binding activity decrease with drug treatment in responsive cells [30] resulting in decreased optical redox ratios and NAD(P)H τ_m_, and increased FAD τ_m_. This leads to a decrease in the OMI index (OMI index = redox ratio + NAD(P)H τ_m_ − FAD τ_m_) with drug treatment in responsive cells [31]. Additionally, changes in NAD(P)H lifetimes correlate with changes in intracellular oxygen levels in organoids [32] while changes in the NAD(P)H/FAD ratio relate to changes in oxygen consumption [33] and glutamine consumption [34]. Previous studies have validated that OMI of PDCOs captures early metabolic changes at the single cell level that predict later treatment response in vivo [29,35,36]. Here, OMI of GEP-NET PDCOs was used to show that the novel combination of ABT263 (Bcl-2 and Bcl-xL inhibitor) and everolimus (mTORC1 inhibitor) causes response in more GEP-NET PDCOs than either agent alone. This study is the first to use OMI to measure drug response in a PDCO model of gastroenteropancreatic neuroendocrine tumors. OMI identifies treatment response where other methods such as PDCO diameter and proliferative rate fail due to the low proliferation rate of this disease. These novel methods of GEP-NET drug screening could streamline drug development for GEP-NET patients who are in great need of new therapies.

## 2. Materials and Methods

### 2.1. STC-1 Cell Line Experiments

STC-1 mouse neuroendocrine cells (ATCC) were cultured in DMEM + 10% FBS + 1% Pen-Strep. A dose response curve was performed using a 96 well plate WST-1 assay to determine the proper dose of everolimus (range: 0.1 nM–5 nM) as the cells are a mouse line, grown in 2D, and cell lines have previously been shown to be more sensitive to everolimus [37]. Imaging experiments were performed by plating cells on glass bottom imaging dishes at a concentration of 200 K cells/dish and grown for 24 h prior to treatment. After 24 h, media were removed and replaced with either fresh media (control) or media containing 1 nM everolimus, 250 nM ABT263 or both 1 nM everolimus and 250 nM ABT263. Cells were treated for 72 h prior to imaging. Fluorescence lifetime images were taken on a custom-built inverted multiphoton microscope (Bruker Fluorescence Microscopy, Middleton, WI, USA), as described below. After imaging cells were fixed with 2% paraformaldehyde and immunofluorescence staining was performed as described below. Conjugated antibodies against Ki67 and CC3 (11882S-488 conjugate, and 8172S-594 conjugate, Cell Signaling Technology, Danvers, MA, USA) both at a dilution of 1:50 were applied before coverslips were mounted onto slides with Prolong Gold DAPI mounting media (P36931, Invitrogen, Carlsbad, CA, USA) and sealed with clear nail polish. After 24 h stored at 4 °C in a dark box, the slides were imaged on a Nikon Eclipse Ti2 inverted widefield fluorescence microscope using a 20× air objective (0.75NA), with standard Nikon filters for DAPI, FITC, and Texas Red (DAPI: 375/28ex, 460/60em; FITC: 480/30ex, 535/45em; Texas Red: 560/40ex, 630/60em). For analysis, 6 images were acquired from two separate biological replicates for a total of 12 images analyzed per condition. Colocalization analysis was performed using a custom Cell Profiler pipeline, that identified DAPI objects, Ki67 objects and CC3 objects individually. Then, the number of DAPI objects that also overlapped with Ki67 or CC3 positive stain were counted to calculate the % of total cells staining positive for Ki67 or CC3. 

### 2.2. Cell Isolation and PDCO Culture

All studies involving human tissue were performed with approval from the University of Wisconsin-Madison Institutional Review Board with informed consent from patients. Human tissue from needle biopsy or surgical resection was placed immediately into chilled chelation buffer and placed on ice for up to one hour for transportation from the hospital. The sample was then washed twice by dipping in culture medium, Advanced DMEM/F12 (Invitrogen, Carlsbad, CA, USA) containing 10% fetal bovine serum and 1% pen-strep. The sample was then placed in digestion buffer (5 mL culture medium + 100 µL collagenase + 62.5 µL dispase) and shaken vigorously before incubation at 37 °C and 5% CO_2_. Depending on initial sample size, the sample was incubated a minimum of 5 min and a maximum of 2.5 h until roughly 80% of the tissue was digested. For longer digests, the sample was removed from the incubator and repetitively shaken in 10–20 min intervals. After digestion, the samples were pipetted vigorously to break them up further, spun at 1000 rpm for 5 min, and the pellets produced were washed with cold sterile 1× PBS to remove any residual digestion buffer. Finally, the PBS was aspirated, and the pellet resuspended in ADF_stock_ (DMEM/F12 + 1% pen-strep + 5 mL 100× Glutamax + 5 mL HEPES). The cell suspension was mixed 1:1 with 50 µL of cold Matrigel and 50 µL of Matrigel/cell mixture was pipetted into each well of a pre-warmed 24-well plate. Dishes were incubated for 2–5 min at 37 °C to allow the Matrigel to solidify, then plates were flipped upside-down and incubated for at least 20 min at 37 °C. This allowed the Matrigel to form a hanging drop in which the PDCOs could grow three-dimensionally. After incubation, plates were flipped right side-up and the droplets covered with ADF_feed_ (10 mL ADF_stock_ + 10 µL EGF) before being returned to the incubator. PDCOs were fed with fresh ADF_feed_ every 2–3 days. 

PDCOs were re-plated 1–2 times after initial culture of 2–4 weeks and maintained in culture for at least one month to ensure viability prior to in vitro assays. PDCOs were re-plated by removing medium and fresh cold medium was used to break up the Matrigel and collect the spheres. If sphere number was low, spheres were pelleted. Spheres were then combined in a 1:1 mixture with fresh Matrigel and plated. GEP-NET tumors are characterized by slow growth, so our methods aimed to maintain this slow growth for clinical relevance. Therefore, PDCOs were re-plated, not passaged, as they do not proliferate enough to repopulate new dishes. The goal of re-plating was to transfer the PDCOs to fresh Matrigel and onto imaging dishes to perform the studies, not to passage cultures to increase the number of PDCOs. GEP-NET PDCOs were maintained in culture for 1–2 months in these studies. The low proliferation rate and loss of material with re-plating limits longer term culture of GEP-NET PDCOs. Additionally, the low proliferation rate of the GEP-NET PDCOs resulted in poor recovery from freezing, and we were not successful with cryopreservation of these lines.

### 2.3. PDCO Diameter Measurements

PDCOs were plated in 24-well culture plates and allowed to grow for one day. Images were taken on a Nikon Ti-S inverted microscope using a 4× objective (day 1). After imaging, PDCOs were fed and allowed to grow for an additional six days. Day 7 images were acquired, followed by additional feeding and growth for 7 more days, at which point day 14 images were acquired. The images were analyzed using ImageJ to determine the relative change in median organoid diameter. All experiments were completed in triplicate, and the number of organoids measured per patient ranged from 13–101 (Table S1). There was no significant difference in PDCO diameter change between days 7 and 14 (*p* > 0.05).

### 2.4. Multiphoton Imaging In Vitro Studies

PDCOs grew four days prior to treatment. There is some difference in the organoid diameter between patient samples at beginning of treatment, but all PDCOs had a diameter of less than 200 mm. For treatment, the regular media were replaced with media containing physiologically relevant doses of the different treatments (250 nM ABT263 and 200 nM everolimus) [38,39,40,41]. PDCOs were kept in treatment media for 72 h prior to imaging. The media conditions in which the PDCOs are maintained contain supra-physiological concentrations of pyruvate, glucose and other key metabolic substrates, to avoid the confounds of limited substrate availability [32]. Fluorescence lifetime images were taken on a custom-built inverted multiphoton microscope (Bruker Fluorescence Microscopy, Middleton, WI, USA), as previously described [35,42]. Briefly, the system consists of an ultrafast laser (Spectra Physics, Insight DS-Dual, Milpitas, CA, USA), an inverted microscope (Nikon, Eclipse Ti, Tokyo, Japan), and a 40× water immersion (1.15NA, Nikon) objective. NAD(P)H and FAD images were acquired sequentially for the same field of view using an excitation wavelength of 750 nm and a 440/80 nm emission bandpass filter for NAD(P)H fluorescence, and an excitation wavelength of 890 nm and a 550/100 nm emission bandpass filter for FAD fluorescence. Fluorescence lifetime images were collected using time-correlated single photon counting electronics (SPC-150, Becker and Hickl, Berlin, Germany) and a GaAsP photomultiplier tube (H7422P-40, Hamamatsu Photonics, Hamamatsu, Japan). A pixel dwell time of 4.8 μs was used to acquire 256 × 256 pixel images over 60 s total integration time. The photon count rates were maintained at 1–2 × 10^5^ photons/second to ensure adequate photon observations for lifetime decay fits, and no photobleaching. The instrument response function was measured from second harmonic generation of urea crystals excited at 900 nm, and the full width at half maximum (FWHM) was calculated to be 220 ps. A Fluoresbrite YG microsphere (Polysciences Inc., Warrington, PA, USA) was imaged as a daily standard for fluorescence lifetime. The lifetime decay curves were fit to a single exponential decay and the fluorescence lifetime was measured to be 2.1 ns (*n* = 7), which is consistent with published values [29,42].

### 2.5. Quantification of Fluorescence Lifetime Components

NAD(P)H and FAD fluorescence lifetime images were analyzed using SPCImage software (Becker & Hickl, Berlin, Germany) as previously described [27]. At each pixel, the fluorescence lifetime decay curve was deconvolved with the instrument response function and fit to a two-component exponential decay model, I(t) = α_1_ × exp(−t/τ_1_) + α_2_ × exp(−t/τ_2_) + C. In this model, I(t) is the fluorescence intensity at time *t* after the laser excitation pulse, *α* represents the fractional contribution from each component, *C* accounts for background light, and *τ* represents the fluorescence lifetime of each component [27,43]. A two-component model was used because both NAD(P)H and FAD can exist in two conformational states, bound or unbound [25,28]. The mean lifetime (τ_m_) of both NAD(P)H and FAD were calculated as τ_m_ = α_1_τ_1_ + α_2_τ_2_. The total intensity was calculated from the lifetime data by summing the photons detected at each pixel in the image. The intensity of NAD(P)H was then divided by the intensity of FAD for each pixel to calculate the optical redox ratio. 

As previously described, an automated cell segmentation pipeline was created in Cell Profiler [44] and applied to NAD(P)H intensity images. Briefly, pixels belonging to nuclear regions were defined by an intensity threshold between background and cell cytoplasm intensity and the resulting round objects were stored as a mask. Cells were identified by propagating out from each nucleus. An Otsu Global threshold was used to improve the propagation and prevent it from continuing into background pixels. Cell cytoplasm was defined as the cell border minus the nuclei. Values for τ_m,_ τ_1_, τ_2_, α_1,_ and intensity of NAD(P)H and FAD, as well as the optical redox ratio, were measured for each cell cytoplasm. These data were used to calculate the OMI index for each cell using a linear combination of the normalized redox ratio, NAD(P)H τ_m_, and FAD τ_m_, with coefficients of (1, 1, −1), respectively. For each variable, values for all cells within a treatment group within a patient were pooled together, the mean and 95% confidence interval for each OMI variable are reported in Table S1. The number of PDCOs analyzed per patient for the OMI analyses are included in Table S2, along with the number of cells per patient for single-cell OMI analysis.

### 2.6. Subpopulation Analysis

Over 100 cells per patient per treatment condition are required for robust estimation of cell sub-populations [45], and three patients met this criteria (Patients 1, 6, 7). Histograms of number of cells vs. OMI index were generated for each treatment condition in these three patients. The histograms were then fit to a Gaussian mixture distribution model containing one or two components by using an iterative expectation maximization algorithm. Each component represents a distinct population of cells, with an upper limit of three populations to avoid over-fitting. The goodness of fit for each model was evaluated using the Akaike information criterion [46], which also penalizes over-fitting. This process was repeated 100 times and only the model with the best fit was implemented. For easy visual comparison, the distributions shown have been normalized to have equal areas under the curve. 

The heterogeneity index was previously validated to predict tumor response in vivo and in tumor organoids [47]. Here we present a modified version, the weighted heterogeneity index (wH-index), which also takes into account the standard deviations of all subpopulations [48,49].

$$wH-index=\sum \left(1-p_{i}\ln\left(p_{i}+1\right)\right)\times \left(\sigma_{i}+d_{i}\right)$$

where *i* represents each subpopulation, *d* represents the distance between the median of each subpopulation and the median of the entire distribution, *p* represents the proportion of all cells belonging to that subpopulation, and σ is the standard deviation of the subpopulation. 

### 2.7. Statistical Analysis 

Differences in OMI index between treatment and control organoids were tested using a one-way ANOVA of control vs. treatment, with a *p*-value < 0.05 defined as significant. Treatment effect size was calculated using Glass’s Δ [50,51]. Glass’s Δ was used because comparisons of large sample sizes, such as those acquired here with single cell measurements, almost always pass traditional significance tests unless the population effect size is near zero. For Glass’s Δ measurements, a cutoff of 0.75 was chosen to indicate significant effect size based on previous studies [36,49]. 

### 2.8. Histologic Processing and Staining

PDCOs were re-plated 1–2 times and maintained in culture for at least one month before plating on glass 22 mm circular coverslips for histologic processing and staining. Note that the proliferation rate of these PDCOs was insufficient to repopulate the cultures, so re-plating was used to refresh the cultures. Histologic analysis was not performed at later time points due to the low proliferation rates of the cultures. PDCOs were fixed in 2% paraformaldehyde for 15 min. The paraformaldehyde was removed, and the fixed PDCOs were rinsed in PBS followed by storage in PBS at 4 °C until staining was performed. H&E staining was performed as previously described [52]. Briefly, the fixed PDCOs were moved through hematoxylin, water, acid alcohol, water, saturated lithium carbonate, water, 70% alcohol, alcoholic eosin, followed with an ethanol dehydration series and xylenes before being mounted on a blank slide for imaging. PDCO immunohistochemistry (IHC) was performed by treating the glass coverslips containing fixed spheres with Peroxidazed (PX968H, Biocare Medical, Pacheco, CA, USA) and washing in PBS with 0.05% Tween20 (PBST) before placing them into a humidity chamber and blocking with 5% bovine serum albumin in Tris Buffered Saline (TBS) with 0.05% Tween20 for one hour at room temperature. Coverslips were then washed in PBST and primary antibody was applied before incubation at 4 °C (overnight for Chromogranin A (ab15160, Abcam, Cambridge, UK) and 10 min for synaptophysin (AB9272, Burlington, MA, USA)). Chromogranin A and synaptophysin antibodies were used at dilutions of 1:400 and 1:300, respectively. After incubation, coverslips were rinsed in PBST and secondary antibody (RHRP520H, Biocare Medical, Pacheco, CA, USA) was applied for 30 min. Coverslips were washed in PBST before visualization with DAB chromogen. Samples were counterstained with hematoxylin and subjected to an ethanol dehydration and xylene treatment prior to mounting on slides for imaging. PDCO immunofluorescence (IF) was performed as previously described [52]. A conjugated antibody against Ki67 (11882S, 488 conjugate, Cell Signaling Technology, Danvers, MA, USA) at a dilution of 1:50 was applied before coverslips were mounted onto slides with Prolong Gold DAPI mounting media (P36931, Invitrogen, Carlsbad, CA, USA) and sealed with clear nail polish. H&E as well as Ki67 staining was attempted on all lines (successful in patient lines 1, 3, and 6 for H&E and patient lines 1, 2, and 3 for Ki67), while chromogranin A and synaptophysin staining was completed on 2 lines (patients 1 and 3).

Tissue IHC and IF were performed as previously described [53] using the same antibodies listed above in the PDCO IHC description. Tissue sections collected were stained as negative controls, and no non-specific staining was observed.

## 3. Results 

### 3.1. Optical Metabolic Imaging (OMI) Compared with Standard Measurements of Response

**Figure 1:**
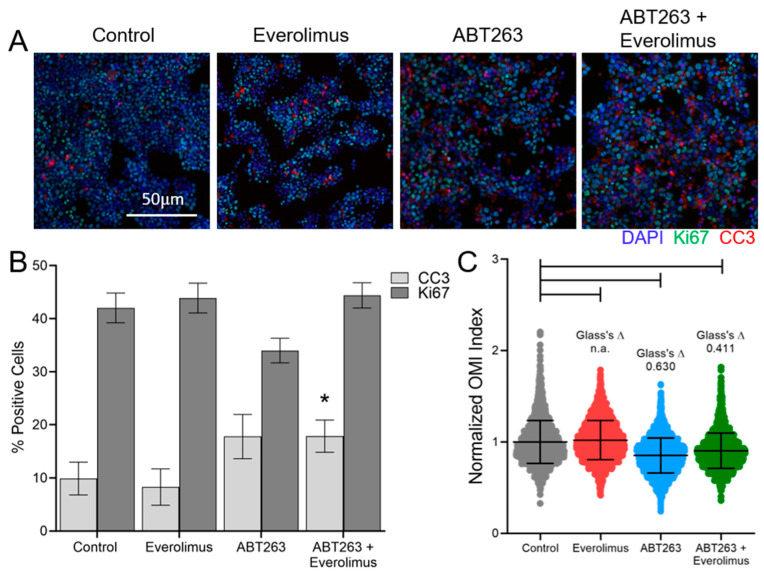
ABT263 and everolimus combination therapy increases cell death and decreases optical metabolic imaging (OMI) index. (**A**) Representative images of STC-1 cells stained for Ki67 (green) and CC3 (red), with DAPI stained nuclei (blue), (**B**) Quantified results from the stain show the number of DAPI cells that were also positive for Ki67 or CC3. There was a significant increase in the percent positive CC3 cells with combination treatment vs. control; * indicates *p* < 0.05, using an ANOVA (*n* = 24 regions of interest), (**C**) Quantified OMI index [linear combination of NAD(P)H mean lifetime (τ_m_), FAD τ_m_, and redox ratio with the coefficients [1, 1, −1], respectively from STC-1 cells treated with ABT263 and everolimus. Redox Ratio is defined as NAD(P)H intensity/FAD Intensity. Bars indicate *p* < 0.01 from an ANOVA (*n* = 2500 cells). Glass’s delta (Δ) values are also shown for treatment vs. control conditions, n.a. indicates non-applicable Glass Δ due to increase in OMI index (decrease in OMI index indicates drug response, increase or no change in OMI index indicates non-response [20]).

STC-1 cells (mouse NET) were used to determine the effect of ABT263 and everolimus treatment in NET cells using standard measurements of response. Immunofluorescence with DAPI, Ki67, and Cleaved Caspase-3 (CC3), an apoptosis marker, revealed that STC-1 cells have a high baseline proliferation rate of around 40% Ki67 positive cells (Figure 1A,B). This emphasizes the need for new models to investigate NET treatments as the percent of Ki67 positive cells for NET patients was much lower, less than 13.5%, for all patient samples in our study (Table 1). However, this mouse NET cell line served as one of the few available models for NET studies and provided relevant material for validation purposes. No significant difference in the percent of CC3 positive cells was identified with either of the single treatments alone (Figure 1B). The combination treatment did lead to a significant increase in the percent of CC3 positive cells compared to control (Figure 1B). 

The OMI index significantly decreased in STC-1 cells treated with ABT263 plus everolimus compared to control (Figure 1C), consistent with drug response. Additionally, the OMI index decreased with ABT263 treatment alone (Figure 1C), and the percent of CC3 positive cells increased with ABT263 treatment alone but did not reach statistical significance (Figure 1B). The increase in OMI index with everolimus alone (Figure 1C) indicates a lack of response. OMI measures response on a single cell level so the sample sizes are large for each condition. Therefore, treatment effect size was calculated using Glass’s Δ, which provides a more conservative estimation of significance. Previous work has shown that a Glass’s Δ of greater than 0.75 corresponds with a significant effect based on other metrics [36]. Overall, these parallel studies of CC3 stains and OMI indicate that decreases in the OMI index are consistent with increases in apoptosis for NET cells, in agreement with previous studies using other cancer models [49].

### 3.2. Patient-Derived Cancer Oranoids of Well-Differentiated NET

**Figure 2:**
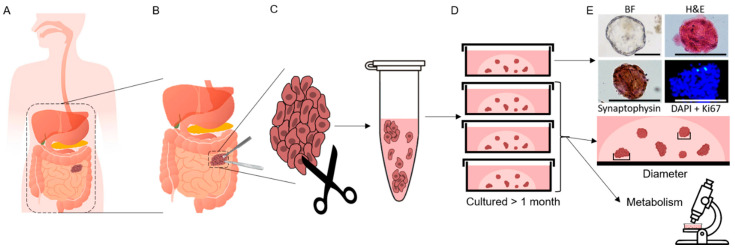
Generation of gastroenteropancreatic neuroendocrine tumors (GEP-NET) patient-derived cancer organoids (PDCOs). (**A**) Patients present with Gastroenteropancreatic neuroendocrine tumors, (**B**) tumor is surgically removed as part of standard treatment, (**C**) a portion of the resected tumor is taken to the lab and digested using dispase and collagenase, (**D**) after the digestion buffer is removed, cells are resuspended in culture medium and combined with Matrigel before plating on glass bottom imaging dishes. PDCOs are maintained in culture for at least 1 month, and re-plated prior to experiments. Due to low proliferation rates, GEP-NET PDCOs do not repopulate dishes and are therefore not passaged, (**E**) some control dishes are used for standard staining when enough material is present (Hematoxylin & Eosin, Synaptophysin, DAPI + Ki67; scale bars 100 µm). All other dishes are treated with drugs and monitored for drug response using brightfield imaging of PDCO diameter change and optical metabolic imaging of metabolic response.

PDCOs were generated from surgical specimens or biopsies for all GEP-NET samples (Figure 2). The overall rate of organoid formation was 90% (9 of 10 samples resulted in viable PDCOs). Due to the slow growing nature of GEP-NET PDCOs, cells were re-plated by placing into fresh Matrigel on new dishes, approximately once every 2 weeks. Re-plating maintains the cultures of PDCOs originally generated, as the proliferation rate is too low to repopulate cultures within a meaningful time. Of those samples where re-plating was attempted, 67% maintained viability over more than two re-platings. Due to small sample size and the destructive nature of staining procedures, only some PDCO samples were stained using hematoxylin and eosin (H&E), immunohistochemical stains for the endocrine markers synaptophysin, and chromogranin or Ki67 (a marker of proliferation; Figure 2E). This staining was performed after PDCOs had been maintained in culture for more than 1 month. The clinical characteristics for each of the PDCO samples that were successfully re-plated are shown in Table 1.

**Figure 3:**
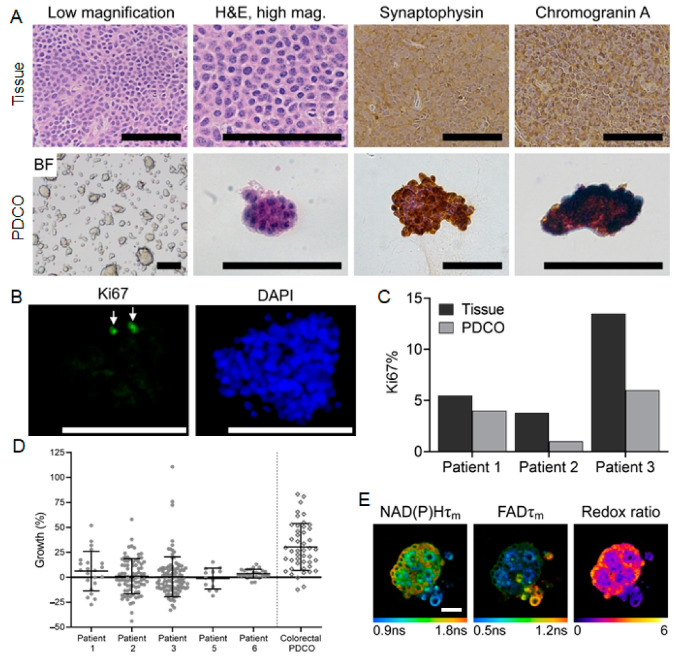
GEP-NET PDCOs maintain key phenotypic characteristics of patient GEP-NETs. (**A**) Comparison of H&E and GEP-NET specific stains, synaptophysin and chromogranin A, between tumor slices and PDCOs from a single patient; scale bars 100 µm, (**B**) Ki67 and DAPI stained PDCOs show slow low percentage of cells with Ki67 staining. Ki67+ cells are marked by white arrow; scale bar 100 µm, (**C**) percent Ki67 positive cells assessed from PDCOs and the patient tissue from which they were derived, (**D**) scatter plots of PDCO growth after 7 days of culture. Growth % = [(day 7 diameter − day 1 diameter)/day 1 diameter] × 100. Each datapoint is one organoid. GEP-NET PDCOs have relatively low growth rates compared to fast growing colorectal cancer (diamonds). See Table S1 for sample sizes, (**E**) optical metabolic imaging of the NAD(P)H mean lifetime (τ_m_), FAD τ_m_, and the Redox Ratio (NAD(P)H intensity/FAD Intensity) for a representative GEP-NET PDCO sample, which indicates that the cells are metabolically active even with low proliferation rates; scale bar 50 µm; ns indicates “nanoseconds”.

The histology of the original patient tumors, by H&E staining, was compared to whole mounts of the resulting PDCOs for a subset of patients. In all cases, the organoid cultures were morphologically similar to the tumors from which they were derived, including a similar well-differentiated morphology represented by a monotonous nuclear appearance with “salt and pepper” chromatin characteristic of neuroendocrine tumors (Figure 3A). Both primary tumors and the PDCOs show immunopositivity for synaptophysin and chromogranin A, which are two immunohistochemical markers for neuroendocrine differentiation (Figure 3A). Synaptophysin is an integral membrane glycoprotein that occurs in presynaptic vesicles, while chromogranin A is protein secreted by neuroendocrine cells [54,55]. 

Ki67 is a marker of cell proliferation and the percent of positive Ki67 cells is slightly decreased in the PDCOs compared to the patient tumor (Figure 3B,C), highlighting an advantage of PDCOs over current GEP-NET cell lines, which typically exhibit Ki67 percentages of 20–90% [13]. GEP-NET PDCOs maintain a percent Ki67 positive cells that more closely match patient tumor proliferation rates than GEP-NET cell lines and are therefore a more relevant model (Figure 3C). Together these data (Figure 3A–C) indicate that GEP-NET PDCOs maintain the histological features and growth rates of the original patient tumors. 

Clinical growth of GEP-NETs is often slow, with samples commonly presenting <20% of cells as Ki-67 positive [56,57]. This slow growth is recapitulated in the untreated control PDCO from five GEP-NET patients in this study (Figure 3D). Changes in diameter for individual organoids over 48 h in untreated GEP-NET PDCOs show distributions centered at zero. A 48-h growth distribution from an untreated colon cancer PDCO line is shown for comparison. These data indicate that a standard measure of drug response in PDCO, organoid diameter, is not suitable for GEP-NET PDCOs due to their slow baseline growth rate. This slow baseline growth also makes another drug response standard measurement, the Ki67 proliferation rate, problematic for GEP-NET PDCOs (Figure 3C). Therefore, alternative methods are needed to monitor treatment response in GEP-NET PDCOs.

### 3.3. OMI of Treatment Response in GEP-NET PDCOs

**Figure 4:**
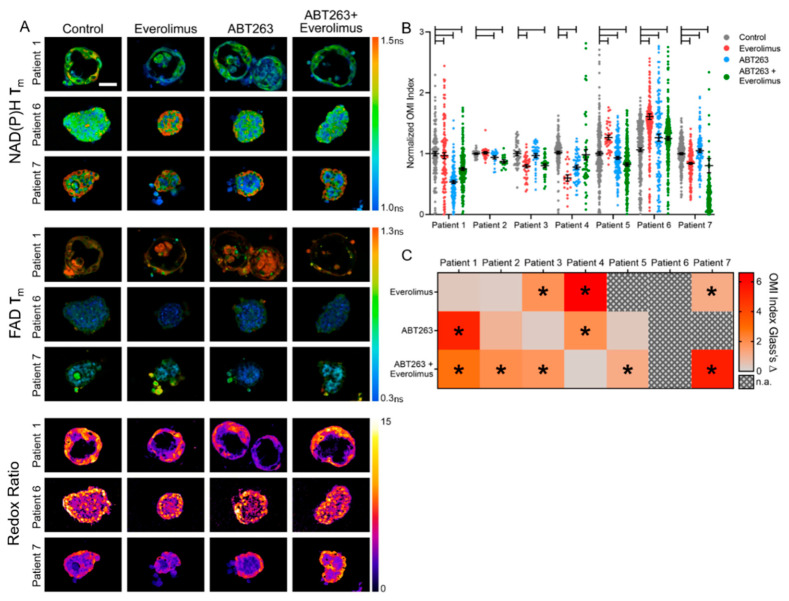
Optical metabolic imaging of drug response in GEP-NET PDCOs. (**A**) Representative images of the NAD(P)H mean lifetime (τ_m_), FAD τ_m_, and the Redox Ratio (NAD(P)H intensity/FAD Intensity) for three PDCO lines; scale bar 50 µm; ns indicates “nanoseconds”, (**B**) OMI Index (linear combination of NAD(P)H τ_m_ FAD τ_m_, and redox ratio with the coefficients [1, 1, −1], respectively) for each patient on a single cell level (each datapoint is a cell), unpaired t-test for each treatment compared to control; bars indicate *p* < 0.01. See Table S1 for sample sizes, (**C**) Glass’s Δ heat map of effect size for the OMI Index of each treatment relative to control; * indicates Glass’s Δ greater than 1.0, n.a. indicates non-applicable Glass Δ due to increase in OMI index (decrease in OMI index indicates drug response, increase or no change in OMI index indicates non-response [20]).

GEP-NET PDCOs are metabolically active, as shown by the fluorescence lifetime of NAD(P)H and FAD as well as the redox ratio (Figure 3E), despite their low proliferation and growth rates. Representative images of separate GEP-NET patients illustrate qualitative differences in PDCO morphology, including hollow (Patient 1) and solid (Patients 3–7) PDCOs (Figure 4A and Figure S1). A decrease in the OMI index is indicative of a metabolic response, whereas an increase in OMI index is indicative of non-response [29]. An ANOVA of the OMI index of treatment to control shows that four of the seven patient samples metabolically respond to everolimus alone, four respond to ABT263 alone, and five respond to the combination therapy (Figure 4B). OMI measures response on a single cell level so the sample sizes are large for each condition. Therefore, treatment effect size was calculated using Glass’s Δ, which indicates a significant effect, based on other metrics, when Glass’s Δ is greater than 0.75 [36]. Glass’s Δ similarly shows that five out of the seven patients metabolically respond to the combination treatment of ABT263+everolimus compared to control (Glass’s Δ > 0.75) (Figure 4C). This is subjective evidence for the promise of this drug combination in GEP-NET patients, which is supported by previous studies indicating synergy of ABT263 and everolimus in leukemia [9]. Importantly, some lines (Patients 2 and 5) have a large effect size only with the combination therapy, while others (Patients 1, 3 and 7) have a large effect size with both combination therapy and with single agent therapies (Figure 4C). Furthermore, one line (Patient 4) shows a large effect size with the single agent therapies alone and does not respond to the combination therapy (Figure 4C). This highlights the heterogeneity in response across patients and indicates a need for a patient-specific drug screen.

### 3.4. OMI Quantifies Single Cell Metabolic Heterogeneity

**Figure 5:**
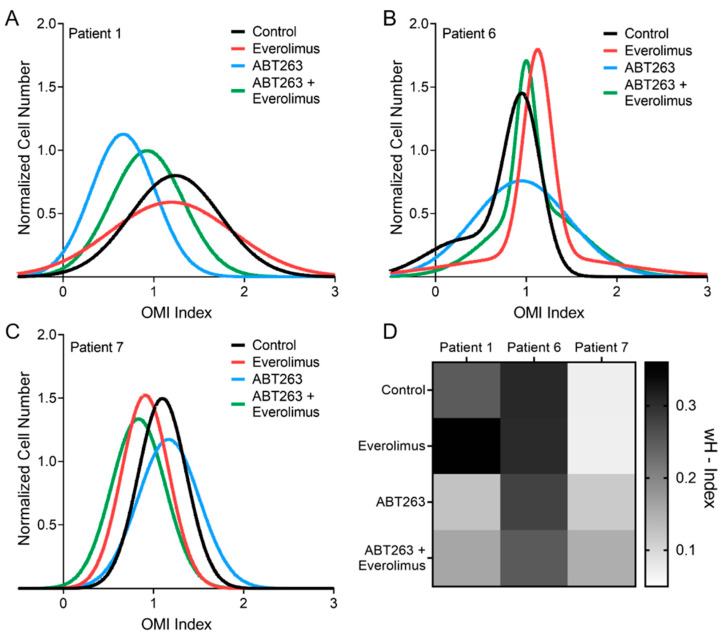
Single-cell subpopulation analysis of the OMI Index (same data shown in Figure 4B) shows differences in heterogeneity between three patients due to treatment. Single cell distributions were fit to a Gaussian mixture model to summarize heterogeneity (see Methods), and the resulting Gaussian fits are shown in (**A**–**C**). (**A**) Patient 1 was best fit with a single Gaussian for all conditions, with a wide variance for everolimus treatment alone, (**B**) patient 6 was best fit with two Gaussians for all conditions, indicating high heterogeneity, (**C**) patient 7 was best fit with a single Gaussian for all conditions, with a narrow variance for each condition, (**D**) heat map of the weighted heterogeneity index (wH-index), which accounts for variance in Gaussian fits and the presence of multiple Gaussians to quantify cellular heterogeneity in a sample (see Methods). Patient 1 has more cellular heterogeneity with everolimus treatment compared to the other conditions. Patient 6 has heterogeneous distributions for all conditions due to two distinct populations for each condition. Patient 7 has low cellular heterogeneity consistent with narrow single population distributions.

Population density modeling of single-cell OMI data was used to determine whether subpopulations, defined as single Gaussian distributions, were present in PDCOs for each treatment condition after 72 h (Figure 5). However, only a subset of patient samples were included in this analysis as previous work has shown that robust modeling requires 100+ cells per treatment group and only three of our samples met that criteria [45]. Models do not reveal distinct metabolic subpopulations of response in PDCOs from Patients 1 and 7 (Figure 5A,C). In each of these cases only one homogenous population was found. However, for Patient 6 there are heterogeneous subpopulations present in all treatment conditions including the control (Figure 5B). These distributions can be quantified with a heterogeneity index, which accounts for subpopulations and variance in single-cell distributions [48]. Interestingly, the heterogeneity index is consistent with the overall treatment response in these patients. Patient 1 and 7 have low heterogeneity indices (Figure 5D) and showed response to the combination therapy (Figure 4B). Conversely, PDCOs generated from Patient 6 have a high heterogeneity index (Figure 5D) and were non-responsive to all treatments (Figure 4B). 

### 3.5. OMI Measurements of Inter-Patient Heterogeneity

A heatmap of Z-scores across all OMI variables, patients, and treatments illustrates heterogeneity within and between patients (Figure S2). Most patients show Z-score decreases for NAD(P)H τ_1_, τ_2,_ and τ_m_, as well as an increase in Z-score for NAD(P)H α_1_ (percent of lifetime signal that corresponds with τ_1_) across all treatments, except for the ABT263 and combination treatments for Patient 5. There is greater variability between patients in the Z-scores of FAD lifetime variables, as some patients have a positive Z-score for all treatments while other patients have mostly negative Z-scores (especially Patient 7). Overall, these full data (Figure S2) indicate heterogeneity in NAD(P)H and FAD protein-binding activities that underlie composite variables of drug response (i.e., OMI index).

## 4. Discussion

PDCOs enable drug screens directly on patient cells for streamlined drug development and clinical treatment planning. We established GEP-NET PDCOs that were successfully re-plated for 70% of samples (7/10). These GEP-NET PDCOs maintained proliferation rates similar to the original tumor (Figure 3C) along with synaptophysin and chromogranin A expression that is characteristic of neuroendocrine cells (Figure 3A). The slow growth and low proliferation of GEP-NET PDCOs (Figure 3C,D) presents a challenge for standard measures of PDCO drug response (PDCO diameter measurements, Ki67 staining/proliferative rate). Therefore, we perform the first OMI measurements of drug response in GEP-NET PDCOs. Validation experiments in 2D culture confirm that the OMI index measures drug response that is consistent with cleaved caspase 3 stains of apoptosis, similar to previous studies in other cancer models [48]. However, the OMI index is sensitive to metabolic changes that may indicate more subtle forms of cell stress than apoptosis.

This unique combination of GEP-NET PDCOs and OMI was used to test a new drug combination for GEP-NET patients. Here, OMI was collected at 72 h post treatment to maximize differences due to drug treatment [49]. However, future studies can take advantage of the non-invasive imaging capabilities of OMI to image response across multiple time points. In this study, five out of seven PDCO lines were sensitive to a novel combination of drugs including the standard of care everolimus and a Bcl-2 family inhibitor, ABT263 (Figure 4B). Previous studies have shown that decreases in OMI index with drug treatment in organoids predict subsequent in vivo response in mouse models and patients [29,49,58]. The increase in OMI index in PDCOs that do not respond to treatment is likely due to metabolic adaptations within the cells, potentially in efforts to circumvent cytotoxicity [26,45]. OMI further quantified the metabolic heterogeneity of PDCO lines on a single-cell level (Figure 5). GEP-NET patients often show heterogeneous response to treatment, for example, clinical trials for GEP-NET patients have shown that roughly 40% of treated patients will have disease progression [3]. Similar clinical studies of standard treatments, such as sunitinib, likewise show a large proportion of GEP-NET patients (~30%) with disease progression [59]. This highlights the heterogeneity in response across patients and indicates a need for a patient-specific drug screen. OMI is advantageous because it can measure metabolic response to drug treatment on a single cell level, which is amenable to measurements of heterogeneity in drug response within patients. Importantly, the untreated control PDCOs for Patient 6 exhibited a high metabolic heterogeneity and PDCOs for Patient 6 did not show response to any of the therapies tested (Figure 5D and Figure 4B). This is consistent with previous studies that show cellular metabolic heterogeneity in control PDCOs predicts treatment resistance in the PDCOs [49].

This study was not sufficiently powered to robustly address drug synergy but focused on new methods to maintain GEP-NET PDCOs and establish feasibility of drug response measurements with OMI. Further studies are needed to determine whether GEP-NET PDCO response to the combination of ABT263 and everolimus correlates with patient response. However, previous studies from our group using other cancer types (colon and pancreatic) indicate that OMI of PDCO treatment response often predicts later clinical response [36,49]. Additionally, given the small sample number (7 patients), limited surgical and biopsy sample sizes, the low proliferation rate of these tumors, as well as the destructive nature of many standard evaluation endpoints, only a few correlative measurements were collected (H&E, Ki-67, synaptophysin and chromogranin A expression, diameter change data). This highlights the importance of non-destructive drug response endpoints such as OMI for sample-sparing assessments in PDCOs.

## 5. Conclusions

In conclusion, OMI is an attractive label-free tool to measure metabolic changes due to drug treatment in GEP-NET PDCOs. This is an important development because GEP-NETs have low proliferation rates that make standard measurements of treatment response ineffective. This study is the first to use OMI to measure drug response in a PDCO model of GEP-NETs. We have shown that GEP-NETs can grow in 3D PDCO culture and maintain a phenotype that is similar to in vivo patient characteristics. Furthermore, we measured metabolic changes indicative of treatment response in 5 out of 7 PDCO lines with a novel combination therapy of ABT263 and everolimus. These innovative methods for GEP-NET drug screening could streamline drug development for GEP-NET patients, who are in great need of new therapies.

## Figures and Tables

**Table 1:** Summary of the seven patient derived NET PDCO lines and their clinical characteristics.

Sample	Site of Origin	Differentiation	Grade	Ki67%
Patient 1	Pancreatic tail	Well-differentiated	G2	5.5%
Patient 2	Pancreatic head	Well-differentiated	G2	3.8%
Patient 3	Mesentery	Well-differentiated	G2	13.5%
Patient 4	Ileum	Well-differentiated	G1	<2%
Patient 5	Ileum	Well-differentiated	G2	9.1%
Patient 6	Lymph node	Well-differentiated	G2	5%
Patient 7	Duodenum	Well-differentiated	G2	8.15%

## Data Availability

The data presented in this study are available on request from the corresponding author.

## Supplementary Materials

The following are available online at https://www.mdpi.com/article/10.3390/cancers13081873/s1, Figure S1: Representative images of NAD(P)H and FAD mean lifetime (τm) images for PDCOs from Patients 2–5, Figure S2: Heatmap of z-scores ((treatment-control)/control) for all optical metabolic imaging variables in PDCOs from patients 1–7, Table S1: Mean and 95% confidence interval for all variables collected with optical metabolic imaging, Table S2: number of PDCOs and cells analyzed for all conditions and patients.
