# Supplementary material for: Autofluorescence Imaging of Treatment Response in Neuroendocrine Tumor Organoids
Source: Cancers (Basel). 2021 Apr 14;13(8):1873. doi: 10.3390/cancers13081873 (PMC8070804; doi:10.3390/cancers13081873)
Supplement: Supplementary file 1 [file cancers-13-01873-s001.pdf]

cancers MDPI

Supplementary Material: Autofluorescence Imaging of Treatment Response in Neuroendocrine Tumor Organoids

Amani A. Gillette, Christopher P. Babiarz, Ava R. VanDommelen, Cheri A. Pasch, Linda Clipson, Kristina A. Matkowskyj, Dustin A. Deming and Melissa C. Skala

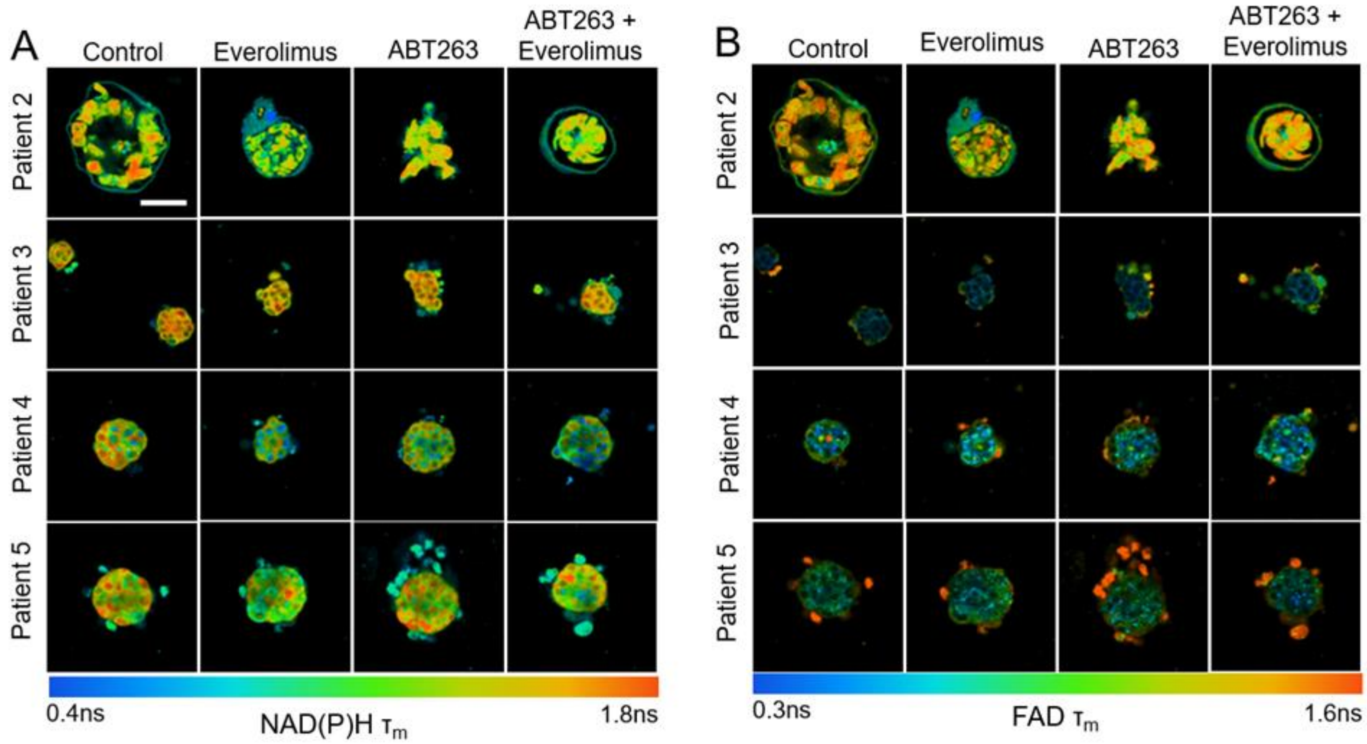

Figure S1. Representative images of NAD(P)H and FAD mean lifetime (τ_m) images for PDCOs from Patients 2-5; scale bar 50μm. ns indicates "nanoseconds".

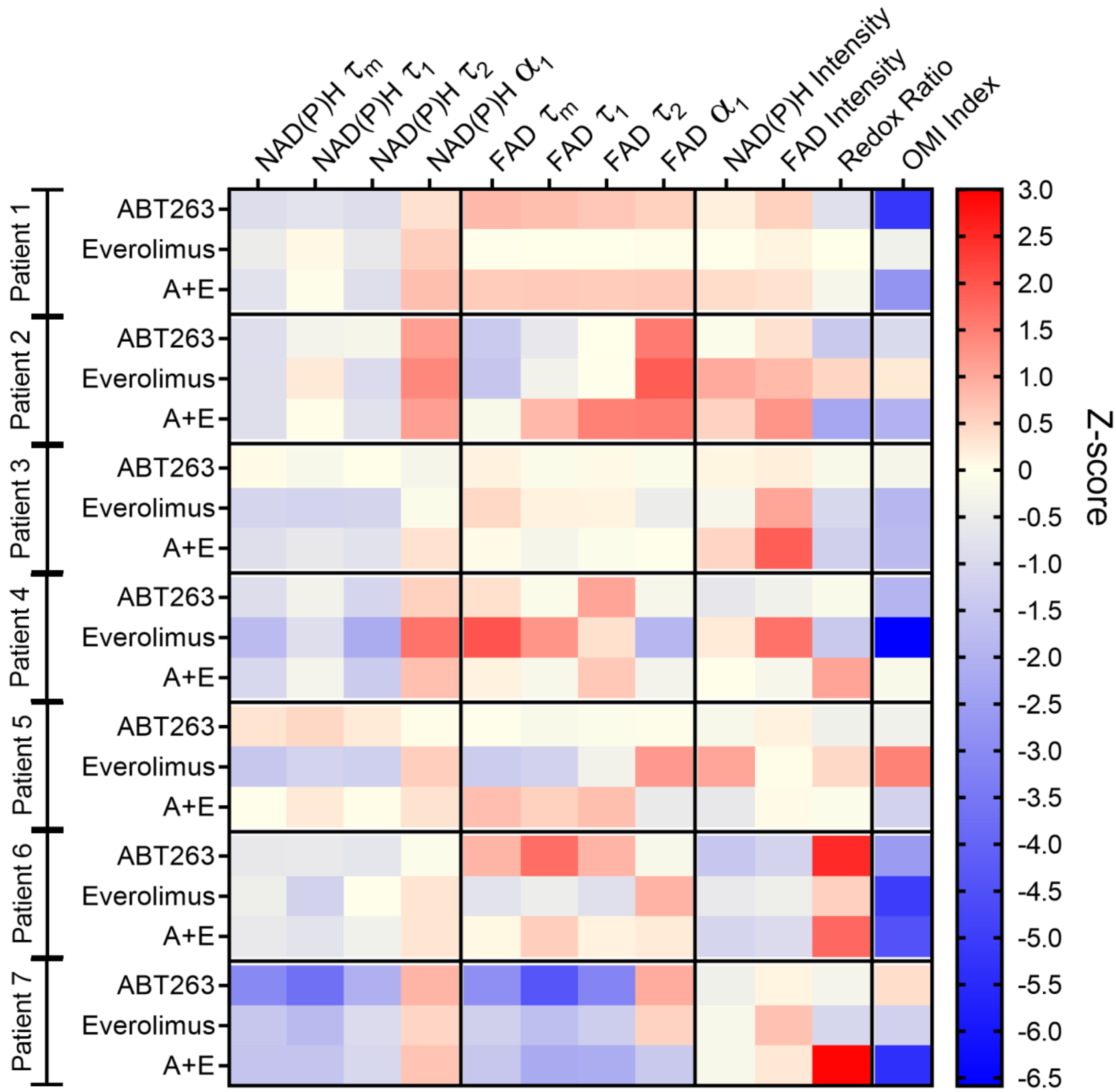

Figure S2. Heatmap of z-scores [(treatment$_{mean}$—control$_{mean}$)/control$_{standard\ deviation}$] for all optical metabolic imaging variables in PDCOs from patients 1-7. These z-scores reflect changes in the treatment condition (row labels, left) compared to control PDCOs from the same patient (patient number, left categories). Heterogeneity in treatment response can be seen in optical metabolic imaging variables (column labels, top) across PDCO lines. "A+E" indicates ABT263 plus everolimus.

Table S1. Mean and 95% confidence interval for all variables collected with optical metabolic imaging. ps, picoseconds; c.u., calibrated units

		NAD(P)H τ_m (ps)		NAD(P)H τ_1 (ps)		NAD(P)H τ_2 (ps)		NAD(P)H α_1 (%)		FAD τ_m (ps)		FAD τ_1 (ps)		FAD τ_2 (ps)		FAD α_1 (%)		NAD(P)H Intensity (c.u.)		FAD Intensity (c.u.)		Normalized Redox Ratio (c.u.)	
Sample	Treatment	Mean	95% CI	Mean	95% CI	Mean	95% CI	Mean	95% CI	Mean	95% CI	Mean	95% CI	Mean	95% CI	Mean	95% CI	Mean	95% CI	Mean	95% CI	Mean	95% CI
Patient 1	Control	1238.06	15.29	626.77	11.15	2499.87	24.86	66.09	0.50	867.85	46.01	364.21	17.76	1923.26	95.38	51.41	2.43	1273.33	70.13	407.83	37.86	1.00	0.05
	ABT263	1154.42	11.28	576.65	8.32	2364.09	18.94	67.15	0.38	1099.04	28.95	446.36	9.22	2306.32	47.78	59.49	1.21	1344.60	59.83	531.38	36.00	0.75	0.04
	Everolimus	1191.58	12.91	631.88	11.70	2405.33	23.90	67.84	0.40	856.97	55.24	363.04	22.24	1913.80	119.51	51.87	3.28	1257.10	61.62	436.89	44.86	1.00	0.07
	ABT263 + Everolimus	1167.21	9.63	622.18	9.33	2371.34	15.74	68.39	0.36	1037.82	24.18	431.90	8.83	2270.72	43.32	60.57	1.18	1445.48	46.06	485.28	31.68	0.93	0.04
Patient 2	Control	1281.81	24.51	650.68	32.81	2139.38	28.27	57.48	0.70	1528.35	15.40	615.81	11.42	2728.54	13.86	56.64	0.52	2772.48	103.90	2480.23	98.75	1.00	0.01
	ABT263	1223.33	37.33	622.55	41.13	2118.29	35.47	59.63	1.37	1469.66	23.99	596.03	22.12	2728.12	26.99	58.87	1.45	2747.50	173.92	2573.23	196.15	0.95	0.04
	Everolimus	1225.44	17.35	672.16	26.74	2065.46	20.69	60.17	0.84	1464.63	12.98	604.97	13.33	2727.28	17.30	59.38	0.75	3056.82	95.25	2699.85	113.35	1.02	0.04
	ABT263 + Everolimus	1225.44	19.74	652.87	29.71	2079.62	23.81	59.64	1.01	1520.94	17.07	641.78	15.45	2784.97	16.75	58.81	0.94	2920.82	155.54	2819.20	136.35	0.91	0.05
Patient 3	Control	1668.27	21.98	836.20	18.79	2811.29	26.56	57.64	0.51	984.75	20.83	390.25	7.67	2415.09	27.66	70.11	0.60	2247.20	66.06	615.81	25.73	1.00	0.04
	ABT263	1671.19	23.95	824.57	29.17	2812.06	35.02	57.19	0.65	995.14	18.27	387.54	7.64	2420.20	25.09	69.89	0.61	2268.42	64.24	631.45	34.88	0.98	0.04
	Everolimus	1588.36	22.35	764.28	21.92	2712.22	24.50	57.44	0.56	1015.72	21.06	393.53	7.66	2426.58	24.81	69.12	0.68	2195.82	70.03	704.33	33.78	0.86	0.04
	ABT263 + Everolimus	1607.34	26.79	798.41	21.85	2745.44	31.89	58.21	0.68	987.49	18.81	383.68	6.69	2406.53	23.56	70.01	0.65	2350.59	70.76	776.77	45.14	0.84	0.04
Patient 4	Control	1139.64	25.54	403.01	9.87	2435.45	25.05	63.45	0.64	414.19	16.93	164.96	14.49	1393.99	22.63	77.87	1.09	1998.95	54.73	1550.77	54.48	1.00	0.03
	ABT263	1005.85	27.08	382.46	13.10	2275.34	32.63	65.43	1.27	449.10	17.46	154.89	5.23	1535.70	50.89	76.35	1.09	1799.18	82.14	1433.61	88.62	0.98	0.06
	Everolimus	872.22	64.48	353.36	21.99	2114.63	67.23	69.59	2.76	613.90	90.74	271.43	85.84	1440.32	70.36	65.72	5.11	2073.10	169.78	2078.93	280.46	0.76	0.09
	ABT263 + Everolimus	982.11	40.02	385.23	16.28	2236.96	45.53	66.20	1.47	428.27	16.10	149.16	6.75	1480.09	48.07	75.80	1.49	2000.14	91.24	1473.20	128.45	1.18	0.18
Patient 5	Control	1411.11	23.76	641.71	19.78	2694.89	31.09	60.82	0.60	712.63	23.71	296.24	15.14	1807.60	25.06	70.64	0.81	1896.37	48.78	931.95	37.42	1.00	0.04
	ABT263	1471.25	35.37	716.11	32.72	2753.49	48.32	60.90	0.72	705.03	27.77	275.45	14.84	1787.94	30.52	70.25	1.07	1820.98	78.99	977.60	43.04	0.88	0.03
	Everolimus	1132.41	38.06	456.41	19.94	2378.85	39.13	63.56	1.51	459.33	15.74	153.93	4.13	1737.24	39.66	78.44	1.73	2309.18	122.85	939.53	61.60	1.14	0.06
	ABT263 + Everolimus	1402.25	36.29	679.93	26.61	2699.55	45.21	62.36	0.78	857.20	35.26	362.33	22.24	1959.30	62.40	67.08	1.05	1656.18	82.86	945.76	87.01	0.97	0.06
Patient 6	Control	1147.63	10.60	444.54	4.37	2419.76	16.74	63.34	0.49	301.43	13.09	109.90	5.21	1632.83	26.08	88.15	0.39	1941.77	44.04	528.68	27.42	1.00	0.04
	ABT263	1094.02	14.69	423.57	6.55	2323.20	23.65	62.95	0.83	396.05	25.08	184.50	15.96	1824.26	53.00	87.44	0.58	1396.95	128.67	267.47	36.99	1.73	0.10
	Everolimus	1108.49	11.51	402.09	5.61	2412.86	10.04	64.59	0.37	222.07	11.10	89.66	4.47	1457.96	23.63	91.09	0.37	1726.16	44.06	426.04	23.26	1.16	0.07
	ABT263 + Everolimus	1097.15	8.14	418.12	3.44	2367.90	9.72	64.50	0.38	310.01	11.76	134.62	6.60	1662.41	24.84	88.93	0.32	1541.87	59.14	310.36	18.68	1.52	0.06
Patient 7	Control	1324.99	14.34	540.34	6.94	2617.39	20.51	59.70	0.60	730.50	11.95	301.56	3.82	2078.79	18.66	73.77	0.63	1031.76	24.99	579.68	22.09	1.00	0.03
	ABT263	996.77	17.09	348.65	6.37	2302.28	33.04	63.58	1.04	468.09	13.30	175.72	3.07	1633.81	24.75	78.33	0.89	954.84	35.44	598.73	32.03	0.94	0.04
	Everolimus	1168.22	23.97	447.85	11.39	2472.24	31.76	61.82	0.63	621.11	17.63	253.58	7.19	1896.86	31.44	76.16	0.59	995.70	32.26	696.21	26.32	0.80	0.02
	ABT263 + Everolimus	1160.68	22.58	460.30	13.01	2456.02	30.68	62.80	0.59	600.15	34.74	238.70	14.01	1777.86	89.61	67.26	3.24	995.49	30.04	623.84	39.96	1.56	0.26

Table S2. Number of PDCOs and cells analyzed for all conditions and patients. "Diameter" – Number of PDCOs (#PDCO) measured for brightfield diameter measurements in control conditions only. Number of PDCOs (#PDCO) and number of cells (#Cells) are also provided for each treatment condition and patient for the analysis of optical metabolic imaging (OMI).

Sample	Diameter	OMI							
	Control	Control		Everolimus		ABT263		ABT263 + Everolimus	
	#PDCO	#PDCO	#Cells	#PDCO	#Cells	#PDCO	#Cells	#PDCO	#Cells
Patient 1	22	12	146	13	120	12	135	13	164
Patient 2	80	3	29	3	25	3	19	3	14
Patient 3	101	5	42	5	41	5	38	6	33
Patient 4	NA	5	133	5	22	5	56	5	49
Patient 5	13	13	251	7	37	12	148	16	191
Patient 6	26	13	267	11	351	10	132	12	349
Patient 7	NA	20	214	18	190	13	114	18	175
Colorectal	46								
